# Simian adenoviruses as vaccine vectors

**DOI:** 10.2217/fvl-2016-0070

**Published:** 2016-09-15

**Authors:** Susan J Morris, Sarah Sebastian, Alexandra J Spencer, Sarah C Gilbert

**Affiliations:** 1 Jenner Institute, ORCRB, University of Oxford, Off Roosevelt Drive, Headington, Oxford, OX3 7DQ, UK

**Keywords:** BAC recombineering, chimpanzee adenoviruses, clinical trials, simian adenoviruses, vector vaccine

## Abstract

Replication incompetent human adenovirus serotype 5 (HAdV-C5) has been extensively used as a delivery vehicle for gene therapy proteins and infectious disease antigens. These vectors infect replicating and nonreplicating cells, have a broad tissue tropism, elicit high immune responses and are easily purified to high titers. However, the utility of HAdV-C5 vectors as potential vaccines is limited due to pre-existing immunity within the human population that significantly reduces the immunogenicity of HAdV-C5 vaccines. In recent years, adenovirus vaccine development has focused on simian-derived adenoviral vectors, which have the desirable vector characteristics of HAdV-C5 but with negligible seroprevalence in the human population. Here, we discuss recent advances in simian adenovirus vaccine vector development and evaluate current research specifically focusing on clinical trial data.

Replication incompetent human adenovirus serotype 5 (HAdV-C5) has been extensively used as a delivery vehicle for gene therapy proteins and infectious disease antigens. However, these vaccine vectors are limited within the clinical setting due to the high seroprevalence, 40–45% in the USA and up to 90% in residents of sub-Saharan Africa, to HAdV-C5 within the human population [1–3]. A recent Phase IIb trial (STEP trial) of an HAdV-C5 vaccine expressing antigens of HIV-1 was abruptly halted due to lack of efficacy [4] and the subsequent finding that participants in one subgroup with circulating neutralizing antibodies to HAdV-C5 prior to vaccination showed a nonsignificant increase in acquiring HIV infection [5,6]. In light of these studies there has been a growing interest in generating vaccines from rare human adenovirus serotypes and nonhuman adenovirus serotypes, which have negligible seroprevalence in the human population [7,8]. Leading nonhuman adenovirus candidates include vectors derived from simian adenoviruses (SAds) [9–11] and in particular those derived from chimpanzee adenoviruses (termed ChAds or AdCs) [12–14]. Although SAds are closely related to human adenoviruses [15] the hypervariable regions of the main immunogen, hexon, are significantly different from HAdV-C5 that they circumvent pre-existing immunity to HAdV-C5. Replication incompetent SAd vaccine vectors lack the essential growth viral transactivator genes encoded by the E1 region and thus vector production requires the expression of E1 proteins in *trans*. SAds and HAdV-C5 share a close homology in the E1 region allowing simian E1 deleted adenovirus vector complementation in cell lines originally derived for complementation of HAdV-C5 E1 deleted vectors [16]. An added benefit is that there is no risk of generating replication competent adenoviruses through recombination events between the SAd genome and the complementing region within the host cell as the E1 flanking regions are different between HAdV-C5 and SAds [12,14].

Vaccine vectors derived from ChAd3, 7, 6, 9, 32, 33, 63 and 68 have been generated [12] and tested in preclinical settings for immunogenicity toward a wide range of pathogens including malaria [17–19], HIV [20,21], influenza virus [22], Ebola [23], SARS [7], hepatitis C [24,25], rabies virus [26] and Rift Valley fever [27]. These vectors have been demonstrated to induce immune responses at very low doses in mice (1–3 × 10^6^ viral particles). Protective immunity in preclinical models was equal to, or greater than, that induced by equivalent HAdV-C5 vectors for ChAd63-derived vaccine vectors [12]. Promising preclinical data have led to the use of SAd vaccine vectors in clinical trials where they have been shown to have a good safety and immunological profile [12].

## • SAd vaccine vector design & development

Key considerations in the design of SAd vectors for use as vaccines are similar to those for HAdV-C5. The vaccine vector must be nonreplicating and unlike adenovirus gene therapy vectors have negligible immune modulatory activity. Hence, SAd vectors lack the E1 region encoding viral transactivator proteins which are essential for virus growth and the E3 region encoding immunomodulatory proteins.

**Figure 1.**
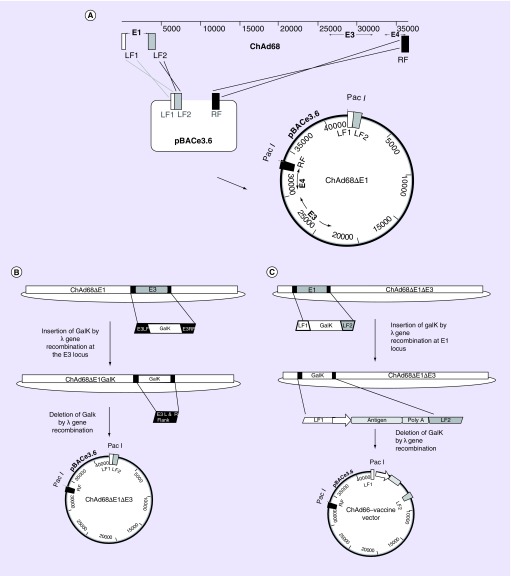
**Generation of a molecular clone of ChAd68.** **(A)** Insertion of ChAd68 genomic DNA into the pBAC ‘rescue vector’ by gap repair. The E1 LF1 and 2 and terminal right hand side region (RF) are amplified from ChAd68 genomic DNA and cloned into pBACe3.6 to produce a BAC adenovirus rescue clone. Recombination occurs between LF1 and LF2 of the isolated ChAd68 genome and the BAC rescue clone and the RF of ChAd68 genome and the BAC rescue clone. The resulting product is a BAC containing an E1 deleted ChAd68 genome. **(B)** Excision of the E3 region of ChAd68 by recombineering. First, the galactokinase gene (*GalK*) is amplified from pGalK using primers containing sequences homologous to the flanking region of E3 (E3LF and E3RF). The E3 region is replaced by the *GalK* gene using λ red recombination. The *GalK* gene is subsequently replaced by a PCR product consisting of E3LF and E3RF, again using λ red recombination. The resulting product is a BAC containing an E1E3-deleted ChAd68 genome. **(C)** Insertion of an antigen cassette at the E1 locus. First, the *GalK* gene is amplified from pGalK using primers containing sequences homologous to the flanking region of E1 (LF1 and LF2). The E1 region is replaced by the *GalK* gene using λ red recombination. The *GalK* gene is subsequently replaced by a PCR product consisting of LF1-antigen-expression cassette-LF2 using λ red recombination. The resulting product is a BAC containing an E1E3-deleted ChAd68 genome with an antigen-expression cassette at the E1 locus. BAC: Bacterial artificial chromosome; ChAd68: Chimpanzee adenovirus 68; LF: Left flanking region; RF: Right flanking region.

**Figure 2.**
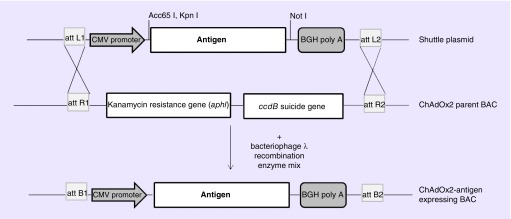
**Insertion of an antigen-expression cassette into adenovirus vector using *att* recombination sites.** A universal cassette expressing a bacteria antibiotic resistance gene and ccdB suicide gene flanked by the specific recombination sequences, *att*R1 and *att*R2 is located at the E1 locus and/or the E3 locus of the BAC-adenovirus genome clone. Shuttle plasmids containing an antigen-expression cassette flanked by specific recombination sites paired with those present in the adenovirus genome (*att*L1/L2) allow site-specific recombination in the presence of an enzyme mixture containing bacteriophage λ integrase, integration host factor and excisionase. BAC: Bacterial artificial chromosome; ChAdOx2: E1/E3 deleted adenovirus vector derived from ChAd68 with a modified E4 region; CMV: Cytomegalovirus.

The advent of bacterial artificial chromosomes (BACs) coupled to bacteriophage λ red recombination (recombineering) technology has facilitated the manipulation of large virus genomes [28]. Using this approach, linear DNA adenovirus genomes isolated from nonhuman primates have been cloned for use as viral vectors. The first stage, following virus isolation and genome sequencing, is either the amplification or artificial synthesis of: two products homologous to the left arm of the genome which flank the E1 region, and; one product, approximately 1000 bp, homologous to the right arm of the genome each incorporating a unique restriction enzyme site for cloning and genome excision for vector production. These fragments are assembled and inserted into a BAC by conventional restriction enzyme cloning. The virus genome is then inserted into the BAC clone by single-step gap repair homologous recombination to generate an E1 deleted viral vector molecular clone (Figure 1A). The recombineering system is then used to allow seamless deletion of the adenovirus E3 immunomodulatory genes. Firstly, the bacterial galactokinase gene (*GalK*) is amplified from the plasmid, pGalK, such that it contains approximately 50 bp homology arms flanking the E3 region, this gene is inserted at the E3 locus of the BAC-rescued adenovirus genome by λ red recombination. Clones are screened for growth on galactose as this phenotype is attributed to the *GalK* gene product. The *GalK* gene is then removed by λ red recombination with a PCR product comprised of the E3 left and right flanking region only (Figure 1)B. Positive clones are selected on 2-deoxygalactose media, which prevents growth of bacteria expressing the *GalK* gene. Further manipulation using λ red recombination firstly to insert the *GalK* gene and then to exchange it for an antigen-expression cassette at the E1 locus completes the engineering of the vaccine vector (Figure 1C) [29]. The linear virus genome is excised from the BAC using unique restriction enzymes, usually PacI or PmeI, and transfected into complementing cells to generate the viral vector. The antigen cassette typically consists of a strong promoter such as the minimal cytomegalovirus (CMV) immediate early promoter, to drive antigen expression, the antigen of interest and a polyadenylation signal. We have generated a molecular toolbox that allows the insertion of any gene easily into a set region within the ChAd genome by inserting universal cassettes expressing a bacteria antibiotic resistance gene flanked by specific recombination sequences, such as *att*R1 and *att*R2, derived from bacteriophage λ (note this system is based on the gateway cloning system from Invitrogen), into our ChAd derived vaccine vectors at the E1 locus and/or the E3 locus. Shuttle plasmids containing an antigen-expression cassette flanked by specific recombination sites paired with those present in the genome (e.g., *att*R1/R2 recombination sequence requires *att*L1/L2 recombination sequence) allow site-specific recombination in the presence of an enzyme mixture containing bacteriophage λ integrase, integration host factor and excisionase (Figure 2).

**Figure 3.**
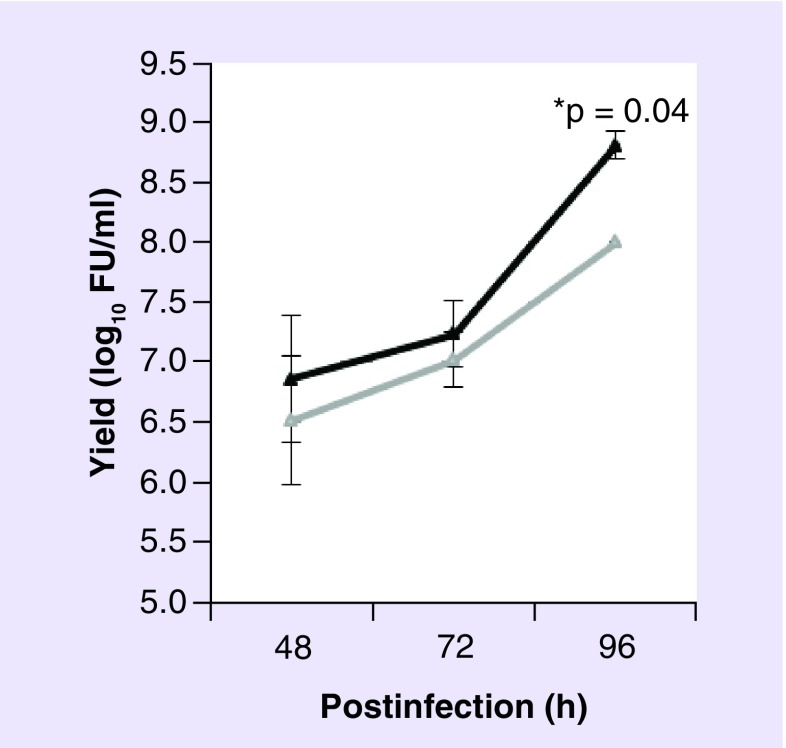
**Growth of ChAdOx2 compared with ChAd68.** E1-complementing HEK293 cells were infected with one multiple of infection of viral vectors ChAdOx2 (black line) or ChAd68 (gray line) each expressing GFP from the E1 locus. Samples were taken at 48 and 96 h postinfection. Virus yield was determined by titration in triplicate on HEK293 cells and GFP-positive cells counted 48-h postinfection. Results are expressed as log_10_ fluorescent units per milliliter from two separate experiments with triplicate titrations for each sample. Student's unpaired *t*-test was used to statistically analyze the results and the mean with standard deviation is depicted. FU: Fluorescent unit; GFP: Green fluorescent protein.

Although the deleted E1 region from SAds is complemented by HAdV-C5 E1 proteins constitutively expressed by human embryonic kidney (HEK293) cells or PerC.6 cells, viral yields vary depending on SAd serotype. High yields of Pan5, ChAd68 (also referred to as Pan 6 or sAd25) and Pan7, all derived from chimpanzees can be obtained from HEK293 cells [16], whereas ChAd1 yields are poor [30]. For virus vectors with poor replication, further genome manipulation has been shown to increase yields. In the case of HAdV-C5, the E4 gene products in particular those from *orf3*, *orf4*, *orf6* and *orf6/7* coordinate their function with the E1 proteins (E1A and E1B 55K) and host cell cofactors to bind, regulate and derepress several cellular functions during viral multiplication [31–36]. Manipulation of the E4 region can therefore be a promising means of increasing virus yields. We have recently described the generation of a chimeric vaccine vector, ChAdOx1, derived from ChAd serotype Y25 engineered by λ red recombination to exchange the native E4 *orf4*, *orf6* and *orf6/7* genes for those from HAdV-C5. This vector showed an increase in hexon protein production from HEK293 cells compared with the ChAd parent virus [37]. Using this approach we have also recently generated ChAdOx2, an E1/E3-deleted vaccine vector derived from ChAd68 with a modified E4 region to increase virus yields in HEK293 cells (Figure 3). Tatsis *et al*. report that exchanging the left and right inverted terminal repeats and packaging signal allowed the complementation of ChAd1 viral vectors [30]. Therefore further modification of SAds, which have previously been neglected as candidates for viral vaccines due to poor productivity in HEK293 cells, could allow complementation and thus expand the ever growing list of SAd viral vaccines.

## • SAd vector engineering to improve immunogenicity

**Figure 4.**
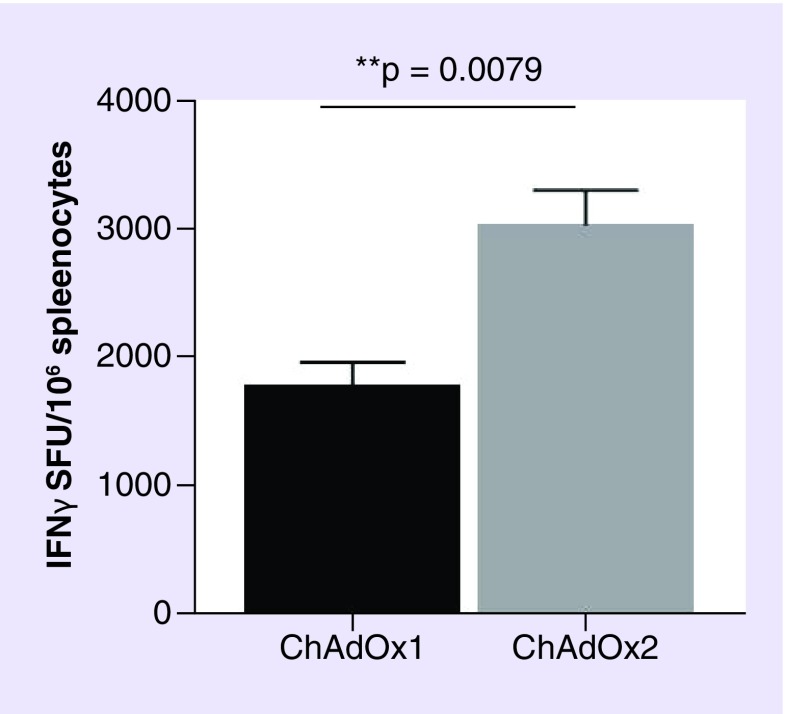
**Immunogenicity of ChAdOx1-eGFP compared with ChAdOx2-eGFP.** Female BALB/c mice (four per group) were injected intramuscularly with 10^8^ infectious units of vector and spleens harvested 2 weeks later to measure the response to GFP by IFN-γ ELISPOT. Results are expressed as SFUs per million splenocytes. Mann–Whitney test was used to statistically analyze the results and the mean with SEM is depicted. GFP: Green fluorescent protein; SFU: Spot-forming unit.

Adenovirus vaccine vectors, regardless of parental origin, can induce humoral, mucosal and cellular immune responses, depending on the route of administration. However, although the T- and B-cell responses elicited are good for most vectors, the level of immunological potency can differ depending on adenovirus vector parental strain/serotype [12,38]. For example, when the two simian vectors ChAdOx1 (derived from Y25) and ChAdOx2 (derived from ChAd68), which both carried a green fluorescent protein (GFP) expression cassette in the E1 locus, were compared, the T-cell response elicited to GFP by IFN-γ ELISpot assay, antigen-specific production of IFN-γ in response to stimulation with the immunodominant GFP peptide was significantly higher in ChAdOx2 mice compared with ChAdOx1 vaccinees (Figure 4). Similar to human adenovirus C serotypes, most SAds use the coxsackie adenovirus receptor, which is present in heart tissue, brain tissue and epithelial and endothelial cells, as the host cell receptor. However, ChAd1 is closely related to human adenoviruses of subgroup B2 and uses CD46, a regulatory protein in the complement system, which is expressed on all human cells except erythrocytes, as the host cell receptor [30]. ChAd1, therefore, has a broad infectivity tropism, allowing it to utilize cells and infection pathways that are not accessible to other Ad serotypes. ChAd7 (sAdV24)-based vectors induce superior protective mucosal immunity in the respiratory tract, particularly following mucosal immunization. This enhanced mucosal immunogenicity could be related to prolonged persistence of the ChAd7 vectors in antigen-presenting cells [39]. Furthermore, differences between vaccine vector immune responses between hosts have also been reported. In mice, after intramuscular immunization, HAdV-C5 based vectors elicited cellular and humoral adaptive responses of higher magnitudes compared with ChAdOx1 and ChAd68 whereas in cattle, cellular and humoral immune responses were at least equivalent, if not higher, in magnitude after ChAdOx1 vaccination compared with HAdV-C5 [40].

Many current studies are driven by a desire to improve SAd vaccine immunogenicity and have focused on producing novel antigen presentation on the viral vaccine surface through engineering of the virus capsid proteins. The SAd hexon protein is the major capsid protein with each viral particle containing 240 copies of the hexon trimer. Antigen epitopes introduced in the hypervariable regions of the hexon protein will be displayed on the virus surface. Zhou *et al*. showed that a linear epitope to the influenza virus M2 protein located within hypervariable region 1, but not hypervariable region 4, of ChAd68 hexon induced a higher antibody response than when M2 was expressed as a transgene at the E1 locus [3]. Introduction of an epitope from coxsackievirus A16 into hypervariable region 1 and an epitope from enterovirus 71 into hypervariable region 2 of ChAd68 generated a bivalent vaccine which elicited a high immune response for hand, foot and mouth disease [41]. The SAd fiber protein has also been a target for improving vaccine immunogenicity. The trimeric fiber protein protrudes from the penton base at each of the 12 vertices of the capsid. The fiber proteins are responsible for host cell receptor binding and thus viral transduction of cells. Engineering of HAdV-C5 fiber has shown that viral vaccine cell tropism can be altered and that insertion of antigen epitopes can elicit an immune response. Fiber modifications of SAds have also been tested. Insertion of the tripeptide arg-gly-asp (RGD) motif into the fiber of ChAd7 expressing an antigen for *Pseudomonas aeruginosa* enhanced mucosal protective immunogenicity by increasing the level of infection of cells expressing high levels of αvβ3 and αvβ5 integrins, such as dendritic cells [42]. Taken together these data show the importance of choosing the correct vector serotype for the desired host and location of immune response, in conjunction with modifications to tropism and method of antigen presentation to ensure the generation of an optimal adenovirus vector vaccine.

## • Clinical trials

Of the many SAds isolated, vectorized and tested in preclinical studies, four have been advanced into clinical vaccine trials to date (Table 1). The first to do so, ChAd63-METRAP, a ChAd63 vector encoding the malaria antigen thrombospondin-related adhesion protein (TRAP) fused to a multiepitope (ME) string containing epitopes from several malaria antigens, was initially used in a Phase I dose and route finding study to assess safety and immunogenicity [43]. In addition to being tested on its own, it was also evaluated as a priming agent in a prime-boost regimen with the modified vaccinia Ankara (MVA) poxviral vector expressing METRAP. In this trial, doses between 1 × 10^8^ and 2 × 10^11^ vp of ChAd63 were found to be safe and elicited high levels of antigen-specific T cells, especially when part of a prime-boost schedule. Based on these encouraging results, the ChAd63 vector (encoding a variety of malaria antigens) has since been used in 21 further Phase I and II malaria vaccine trials, mostly in combination with an MVA boost (reviewed in [44]) but also on its own [45–48], with a protein-in-adjuvant boost [49] or in combination with an MVA vector and the virus like particle vaccine RTS,S (licensed under the name Mosquirix™), which expresses the Asn-Ala-Asn-Pro (NANP) repeat and T-cell epitope sequences from *Plasmodium falciparum* circumsporozoite protein [50,51]. In all of these trials, the ChAd63 vector has consistently proved to be an excellent priming agent for a strong CD8^+^ T-cell response. In addition to malaria vaccine trials, ChAd63 has also been used in clinical studies of HIV vaccines [52]. Here, ChAd63 encoding the HIVconsv immunogen derived from the functionally most conserved regions of the HIV-1 proteosome was tested in combination with MVA or plasmid DNA vaccination, and the prime-boost vaccination regimens were able to induce high frequencies of CD8^+^ T cells specific for the conserved regions of HIV-1. Taking together all clinical trials, the ChAd63 vector has been assessed in more than 1000 individuals to date, including infants and children, and the observed high immunogenicity for both T-cell and antibody responses warrants further development of this vector.

**Table 1.** **Simian adenoviral vectors used in clinical trials.**

**Vector (species isolated from)**	**Classification (group)**	**Trial (phase)**	**Pathogen/disease**	**Ref.**
**PanAd3 (*Pan paniscus*)**	C	I	RSV	[53,54]

**ChAd3 (*Pan troglodytes*)**	C	I, II	Ebola, HCV	[25,55–58]

**ChAd63 (*Pan troglodytes*)**	E	I, II	Malaria, HIV	[43,52]

**ChAdOx1 (modified from *Pan troglodytes* Y25)**	E	I	Influenza A, prostate cancer, tuberculosis	[53,59–60]

Vectors are based on viruses isolated from *Pan paniscus* (bonobo) and *Pan troglodytes* (common chimpanzee).

RSV: Respiratory syncytial virus.

Of equal interest, another chimpanzee adenoviral vector has also made significant progress in a total of ten clinical trials to date: ChAd3 was first used in HCV vaccine trials in heterologous prime-boost schedules together with HAdV-6 [25] or MVA [55]. Both trials recorded durable and broad T-cell responses to the HCV antigen. ChAd3 has also been evaluated in the context of the recent Ebolavirus outbreak. Starting in late 2014, ChAd3 encoding the Ebolavirus glycoprotein (ChAd3-EBO-Z developed by GSK) was fast-tracked into four Phase I trials in the UK, USA, Mali and Uganda [56–57,61], as well as a Phase I/II trial in Switzerland [62]. At doses between 10^10^ and 10^11^ vp, the vaccine showed an acceptable safety profile and significant humoral immunogenicity up to 6 months postvaccination. In fact, antibody-responses 4 weeks after a single dose of ChAd3-EBO-Z were equivalent to those seen in the much publicized Phase III clinical trial of vesicular stomatitis virus-based Ebola vaccine, which showed 100% efficacy [63]. These trials therefore suggest that a single dose of the adenoviral vector may be enough to confer protective efficacy in a ring-vaccination scenario. A large Phase II safety/efficacy study in Liberia was also planned for 2015/16 [58,64], but due to a decline in the incidence of Ebola by the start of the study, efficacy outcomes will likely not be assessable.

The third chimpanzee vector to be tested in the clinic, ChAdOx1, was developed at Oxford University, and is based on the chimpanzee Y25 adenovirus [37]. A Phase I trial of ChAdOx1 encoding the conserved influenza antigens nucleoprotein and matrix protein 1 (ChAdOx1 NP+M1) found high levels of antigen-specific T cells, which were comparable to those elicited previously in trials using the ChAd63 vector encoding malaria antigens [59]. Two further trials involving ChAdOx1 are currently underway, as vaccine candidates against tuberculosis and prostate cancer [53,60].

The most recent simian adenoviral vector to undergo clinical evaluation is PanAd3, which is based on an adenovirus originally isolated from a bonobo [12]. The PanAd3 vector encoding three antigens of the respiratory syncytial virus (RSV) was tested at a dose of 5 × 10^10^ vp in healthy adults, with either intramuscular or intranasal administration, followed by a booster vaccination of MVA encoding the same antigens [54,65]. This prime boost regimen was shown to induce robust RSV-specific T-cell responses post-boost, independent of the route of priming, although as expected, intranasal vaccination with PanAd3 resulted in lower levels of systemic RSV-specific T cells than intramuscular administration. The acceptable safety profile and immunogenicity observed in this trial warrant further clinical investigation of the PanAd3 vector.

## Conclusion & future perspective

SAd viral vaccines, especially those derived from ChAds, are a viable alternative to HAdV-C5-derived viral vaccines. Promising preclinical data have led to the use of ChAd vaccine vectors in clinical trials for a variety of infectious diseases and have shown good safety and immunological profiles in Phase I trials. In the next few years many of these vectors should enter Phase II and III clinical trials providing us with a better understanding of SAd vector immunogenicity in humans. ChAd vectors are also currently being developed as delivery vehicles for antigens against cancers, such as prostate [66] and breast cancer as well as chronic diseases, for example, Crohn's disease. Thus, the use of SAds as a general vaccine vector should become well established in the coming years.

The employment of certain SAdV-based vectors as vaccines may not be suitable for some populations. Although found less frequently than antibodies to HAdV-C5, neutralizing antibodies to some chimpanzee adenovirus serotypes have been detected in humans from sub-Saharan Africa, Brazil and China [2–3,7–8]. Neutralizing antibodies to the vector significantly reduce the specific immune response against the transgene product and thus can be detrimental to the efficacy of a vaccine vector. These data show that an understanding of the immune state of the population to be vaccinated is essential when designing a vaccine vector. Vectors derived from other SAds, rare human adenovirus serotypes and adenoviruses from other species are being developed and may provide additional or alternative vaccine vectors.

A wealth of preclinical data relating to the use of SAd viral vectors as vaccines are available; however, different antigens, virus serotypes and immunization strategies have made it difficult to compare vectors head to head. Future studies need to focus on comparisons of vector backbones, antigen presentation and tropism modification so that information can be collated to allow the establishment of guidelines for the generation of optimal SAd vector vaccines for different hosts and immunological outcomes. These data together with advances in molecular engineering strategies and manufacturing technology will open up the possibility of rapidly generating SAd vector vaccines to combat emerging diseases, such as Ebola and Zika.

**EXECUTIVE SUMMARY**

**Vaccine vectors derived from simian adenoviruses provide a viable alternative to human adenovirus serotype 5 vaccine vectors**

- Simian adenovirus (SAd) vaccine vectors circumvent pre-existing human adenovirus serotype 5 (HAdV-C5) immunity.
- Most SAd vaccine vectors can be grown in HAdV-C5 complementing cell lines.
- Protective immunity in preclinical models for a range of antigens was equal to, or greater than, that induced by equivalent HAdV-C5 vectors for ChAd63-derived vaccine vectors.

**Recombineering technology allows genetic manipulation of the adenovirus genome to generate a range of vaccines with different phenotypic & immunogenic properties**

- SAd viral vaccines are E1E3-deleted viruses.
- Manipulation of the E4 region or inverted terminal repeats increases yields of viral vectors in HEK293 cells.
- Modification of the fiber and hexon proteins provides novel antigen presentation on the surface of SAd viral vectors.
- SAd viral vector immunological potency can differ depending on adenovirus vector parental strain/serotype.
- Parental SAd strain, antigen presentation, the host to be vaccinated and immune response required, for example, mucosal immunogenicity should all be considered in the design of a SAd viral vector.

**ChAd vaccine vectors have been shown to have a good safety & immunological profile in clinical trials**

- ChAd viral vaccines against malaria, Ebola, HIV, influenza, respiratory syncytial virus, HCV, tubercolosis and prostate cancer have all progressed to Phase I clinical trials.
- ChAd viral vectors elicit high levels of antigen-specific T cells and/or neutralizing antibodies when administered as part of a prime boost regimen.
